# Crowdsourced Assessment of Aesthetic Outcomes of Dorsal Preservation Rhinoplasty

**DOI:** 10.1093/asjof/ojae007.095

**Published:** 2024-04-12

**Authors:** Jake Alford, Sean McCleary, Jason Roostaeian

**Affiliations:** University of California - Los Angeles, Los Angeles; UCLA, Los Angeles; David Geffen School of Medicine at UCLA,, Los Angeles, CA

## Abstract

**Goals/Purpose:**

The inherent subjectivity in evaluating aesthetic outcomes presents a unique challenge in assessing rhinoplasty. Crowdsourcing has provided a new metric for objective analysis. The authors designed a study to compare the aesthetic outcomes of dorsal preservation rhinoplasty versus structural rhinoplasty using a reliable and valid crowdsourcing platform. We aim to objectively quantify the relative aesthetic advantages of performing a dorsal preservation technique. Additionally, we aim to demonstrate the efficacy of using crowdsourcing as an efficient and reliable method for evaluating any plastic surgery aesthetic outcome.

**Methods/Technique:**

This retrospective observational study was approved by the IRB at UCLA. A total of 64 patients who had previously undergone rhinoplasty performed by the senior author were included. All surgeries were performed at the UCLA Ronald Reagan Medical Center. Patients with prior nasal surgery or trauma were excluded. All subjects were photographed using standard rhinoplasty views during the pre-and postoperative visits. Frontal and right profile photographs were then cropped in a standardized fashion (Photos, Version 8.0, Apple Inc.) and used for evaluation.

All evaluations and ratings were collected using the HIPPA compliant and encrypted software via a secure online platform (LoveMyDelta, Inc., Philadelphia, PA). All crowd workers were recruited and vetted through this platform, with internal fidelity checks performed. Each crowd worker was shown a profile and frontal view of a patient and asked to score the overall appearance of the nose, the nasal dorsum profile, the symmetry of the dorsal aesthetic lines, and the dorsal contour using a visual analog scale. Scoring was performed using a provided validated visual assessment guide. They were asked whether they believed the subject had undergone rhinoplasty and asked to provide a level of confidence in their guess.

Crowdworkers’ responses were aggregated to allow for high-powered intra-rater analysis. Intra-rater reliability and confidence intervals were calculated. Aggregate data from all raters were used to generate an absolute value of aesthetic state for each cohort. A "delta" was then obtained for each value by comparing the preoperative to the postoperative state to obtain a representative value of the improvement after undergoing surgery. Raters were asked if the patient appeared to have had surgery, and each correct and incorrect response was proportionally weighted based on the raters' confidence in their answer. The delta (difference in preoperative to postoperative aesthetic state) for each parameter for the dorsal and non-dorsal preservation cohorts was calculated using non-paired T-tests to determine significance.

**Results/Complications:**

A total of 64 patients were included for evaluation. The structural rhinoplasty cohort consisted of 34 patients. The dorsal preservation cohort included 30 patients. Both dorsal preservation and non-dorsal preservation rhinoplasty cohorts were associated with improved overall crowdsourced aesthetic outcomes and improved outcomes across all subparameters. Overall, delta *(d)* improved in both dorsal preservation (0.300, 95% CI +/- 0.047) and structural (0.377, 95% CI +/- 0.055) cohorts. When raters were asked to predict whether a patient had surgery, the correlation coefficient between the confidence in the prediction and that prediction being correct was 0.74 (95% CI +/- 0.47 in the structural group, suggesting that a crowd worker was likely to be correct in identifying whether a patient had structural rhinoplasty. However, the correlation coefficient was -0.0554 (95% CI +/- 0.058) in the dorsal preservation group. This suggests the raters were unable to consistently identify which patients had dorsal preservation rhinoplasty with any confidence.

**Conclusion:**

In the ongoing challenge to achieve better aesthetic outcomes in plastic surgery, crowdsourcing is a valuable tool for helping to measure these outcomes. We compared the aesthetic outcomes of dorsal preservation rhinoplasty versus structural rhinoplasty using a reliable and valid crowdsourcing platform. We found that significant improvements in overall aesthetic outcomes were achieved with both techniques, while a more natural “unoperated” outcome was achieved when performing a dorsal preservation technique. With this type of study and assessment tool, we helped to demonstrate the efficacy of using crowdsourcing as an efficient and reliable method for evaluating any plastic surgery aesthetic outcome.

